# A Leg Cuticle Protein Enhances the Resistance of *Anopheles sinensis* Mosquitoes to Deltamethrin

**DOI:** 10.3390/ijms26052182

**Published:** 2025-02-28

**Authors:** Lin Li, Ling Gu, Lei Tu, Si-Jia Deng, Ju-Ping Hu, Zi-Ye Zhang, Ju-Lin Li, Mei-Chun Zhang, Jun Cao, Jian-Xia Tang, Guo-Ding Zhu

**Affiliations:** 1 School of Public Health, Nanjing Medical University, Nanjing 211166, China; lil1221@163.com (L.L.); glgl09@163.com (L.G.); zzy_8882024@163.com (Z.-Y.Z.); caojuncn@hotmail.com (J.C.); 2 National Health Commission Key Laboratory of Parasitic Disease Control and Prevention, Jiangsu Provincial Key Laboratory on Parasite and Vector Control Technology, Jiangsu Provincial Medical Key Laboratory, Jiangsu Institute of Parasitic Diseases, Wuxi 214064, China; dsj780078@163.com (S.-J.D.); hujp622@163.com (J.-P.H.); lijulin301@163.com (J.-L.L.); z02m14c588@aliyun.com (M.-C.Z.); 3 Wuxi School of Medicine, Jiangnan University, Wuxi 214122, China; 15797821819@163.com

**Keywords:** anophelines, pyrethroid resistance, cuticle thickening, decreased penetration, RNAi

## Abstract

Insecticide resistance in mosquitoes has become a severe impediment to global vector control and manifests as decreased insecticide effectiveness. The role of target site mutations and detoxification enzymes as resistance markers has been documented in mosquitoes; however, the emergence of complex resistant phenotypes suggest the occurrence of additional mechanisms. Cuticular proteins (CPs) are key constituents of the insect cuticle, and play critical roles in insect development and insecticide resistance. In this study, via electron microscopy we observed that the leg cuticle thickness in deltamethrin-resistant (DR) *Anopheles sinensis* mosquitoes was significantly greater than that measured in deltamethrin-susceptible (DS) *An. sinensis*. Transcription analysis revealed that cuticle proteins were enriched in the legs, including members of the CPR, CPAP, and CPF families. Further comparisons revealed the specific overexpression of four CP genes in the legs of DR *An. sinensis*; whose expression levels increased after treatment with deltamethrin. The RNAi-mediated silencing of one CP gene, *AsCPF1*, resulted in a significant decrease in the leg cuticle thickness of DR mosquitoes and significantly elevated the mortality rate when exposed to deltamethrin. These findings suggest that alterations in the *An. sinensis* leg cuticle contribute to the insecticide resistance phenotype. *AsCPF1* is thereby a target study molecule for investigation of its mode of action, and broader attention should be paid to the role of mosquito legs in the development of insecticide resistance.

## 1. Introduction

*Anopheles sinensis* is a main vector of malaria and other parasitic diseases in China and other Asian countries [1]. Currently, vector control has proven effective in preventing the transmission of vector-borne diseases, with the utilization of insecticides constituting the principal component of this control strategy [2]. In residential areas, indoor residual spraying (IRS) and insecticide-treated nets (ITNs) have achieved notable success [3]. However, the evolutionary pressure of widespread and prolonged use of insecticides has led to the selection of high levels of resistance in mosquitoes, thus posing a significant challenge to mosquito control efforts [4].

The development of insecticide resistance is complex, with components of both behavioral and physiological resistance. Physiological resistance includes the mutation of insecticide target sites, the increase in detoxification metabolizing enzyme activity, and changes in cuticle structure. Research on mosquito resistance to pyrethroids has focused in part on target-site and metabolic resistance mechanisms [5]. It has been found that the mortality rates of the cockroach *Blattella germanica* and the fruit fly *Bactrocera dorsalis* when exposed to different modes of insecticide application are lower for topical insecticides than for ingested or injected insecticides [6,7]. This may relate to alterations in the internal composition and structure of the cuticle, thus preventing insecticides from binding to their targets or slowing down the rate of insecticide penetration, and consequently providing sufficient time for metabolic enzymes to degrade the insecticides [8]. Therefore, the role of cuticle modification may be overlooked, and more attention should be paid to this mechanism [9].

The cuticle consists of an epicuticle and pre-epidermis, and the pre-epidermis contains mainly cuticle proteins and chitin, whereas the epicuticle is largely composed of lipids [10]. Studies suggest a strong link between cuticle changes in insects and the development of resistance to deltamethrin, which may be related to cuticle thickening and variations in composition [11,12,13,14]. Cuticle proteins (CPs) serve as primary structural constituents of the cuticle, and play important roles in the environmental adaptation and defensive responses of insects. Certain CPs bind to chitin to preserve the integrity of the cuticle, and deletion of related genes can result in alterations in insect morphology and sensitivity to external conditions [9,15,16]. Artificial inhibition of the expression of specific cuticle protein genes in mosquitoes can limit normal cuticle formation, thereby increasing the susceptibility of mosquitoes to insecticides, and this strategy may be useful in improving the effectiveness of existing insecticides [17].

Mosquito legs are principal contacts for the active chemicals of IRS, ITN, and other interventions of adult vector control, making them interfaces for insecticide actions [18]. Gene enrichments have been identified related to neurotransmitters, odor receptors, detoxification metabolism, and cuticle structure in the legs of *Anopheles coluzzii* and *Aedes aegypti*, thus further suggesting the importance of legs in sensing the external environment [19,20]. However, limited research has focused on the role of legs in *An. sinensis* in the development of insecticide resistance, and the related mechanisms remains unclear. To characterize this hypothesis, in this study we analyzed the leg cuticle structure of susceptible and deltamethrin-resistant *An. sinensis*, and we identified and verified the candidate leg-enriched cuticle genes associated with insecticide resistance. This contributes to understanding the development of insecticide resistance attributed to the leg cuticles in mosquitoes.

## 2. Results

### 2.1. Transmission Electron Microscopy (TEM) Analysis of the Tarsus Cuticle of Anopheles sinensis

This study used an *An. sinensis* line which was collected in Huai’an, and the resistance of an F1 generation to deltamethrin was evaluated by an insecticide resistance bioassay. The mortality rate was roughly 13% following exposure to deltamethrin, which indicated a resistant population. The following studies compare this deltamethrin-resistant (DR) *An. sinensis* with a deltamethrin-susceptible (DS) *An. sinensis* mosquito line which is described in the Materials and Methods section.

**Figure 1:**
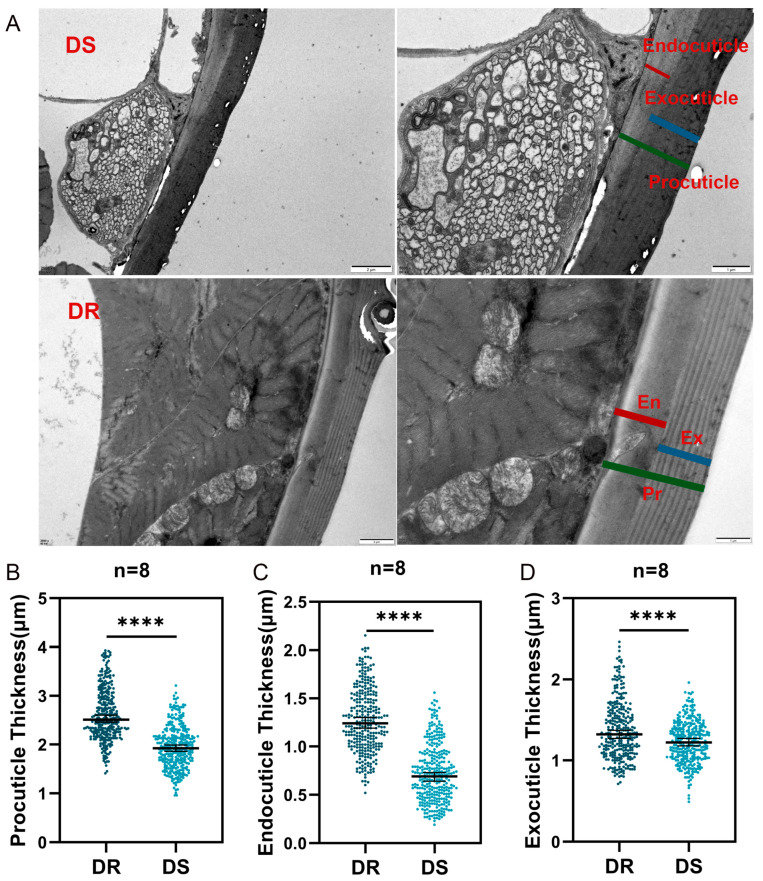
TEM analysis of the thickness of the tarsal cuticle of *An. sinensis* legs. (**A**) Representative TEM images showing cross-sections of the cuticle from the tarsus of *An. sinensis*. (**B**–**D**) The thickness of the cuticle was measured in the cross-section of the tarsus from DS and DR female *An. sinensis*. Each dot represents measurements from individual cuticle locations (each group contains 8 samples, and each sample has 40 points). Asterisks indicate significant differences (Student’s *t*-test, **** *p* < 0.0001).

The leg cuticle is predominantly formed by the procuticle, which is segmented into the exocuticle and the endocuticle (Figure 1A). The procuticle thickness of DR *An. sinensis* (2.62 ± 0.53 μm) was significantly greater than that of DS *An. sinensis* mosquitoes (1.95 ± 0.44 μm) (Figure 1B). There were statistical differences between the endocuticle (1.27 ± 0.33 μm, 0.72 ± 0.30 μm) and exocuticle (1.37 ± 0.36 μm, 1.22 ± 0.26 μm) thickness of the two strains, and the difference in the thickness of the endocuticle appeared to be greater (Figure 1C,D).

### 2.2. Principal Component Analysis (PCA) of Transcriptome Data

**Figure 2:**
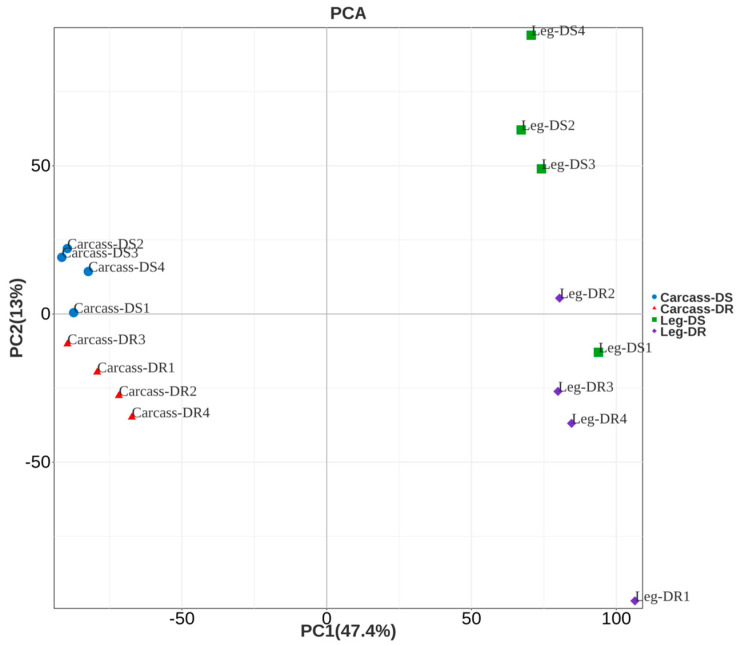
Principal components analysis of the gene expression levels for *An. sinensis* leg and carcass samples. “Carcass” means the parts of the body without legs. The sample types include the carcass of deltamethrin-susceptible *An. sinensis* (Carcass-DS), carcass of deltamethrin-resistant *An. sinensis* (Carcass-DR), leg of deltamethrin-susceptible *An. sinensis* (Leg-DS), and leg of deltamethrin-resistant *An. sinensis* (Leg-DR).

In this study, a comprehensive transcriptomic analysis was conducted to identify cuticle protein genes enriched in the legs of *An. sinensis*, by comparing the legs with the other body parts and examining differences between the legs of DS and DR *An. sinensis*. For transcriptome analysis of insecticide resistance, a dataset was obtained which was composed of 741.2 million clean reads of reverse-transcribed mRNA. The repeatability within the sample group and the discrimination between groups were evaluated by principal component analysis (Figure 2), which showed that the biological replicates of different types of samples had good clustering, and the different samples were clearly distinguished. Among them, there were outliers in the Leg-DS and Leg-DR sample replicates, which were excluded to improve the reliability of subsequent analyses.

### 2.3. Differential Expression Analysis of Cuticle Protein Genes in the Leg of Resistant Anopheles sinensis

Gene Ontology (GO) analysis was initially conducted on differentially expressed transcripts between datasets. Compared to carcasses of *An. sinensis*, legs showed significant enrichment of genes associated with calcium ion binding, fatty-acyl-CoA reductase (alcohol-forming) activity, cilium and cilium assembly. Further comparative analysis between leg types revealed that in DR *An. sinensis*, leg-specific genes were predominantly enriched in heme/iron ion binding, membrane functions and glutathione metabolic processes (Figures S1 and S2).

**Figure 3:**
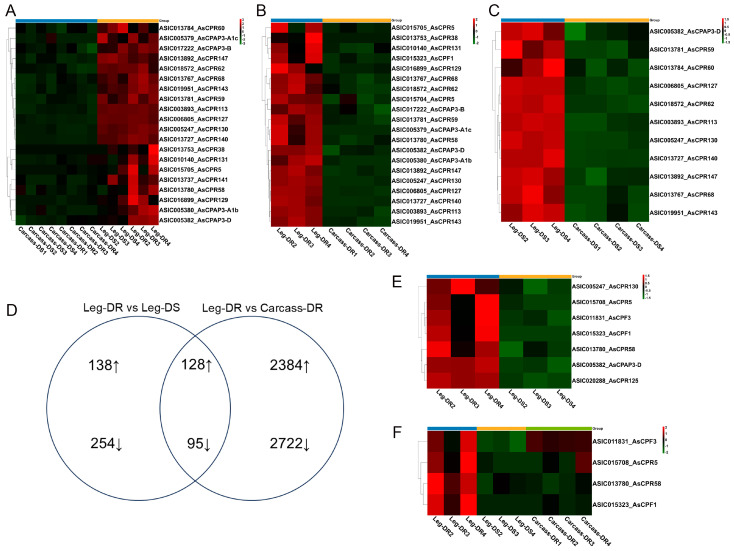
Heat maps showing the expression of cuticle protein genes in different body parts. The heat maps show the log2-fold change (log2FC) value relative to the legs on the red and green rulers. Red indicates overexpression. (**A**) CP genes enriched in leg, compared with carcass. (**B**) CP genes enriched in Leg-DR, compared with Carcass-DR. (**C**) CP genes enriched in Leg-DS, compared with Carcass−DS. (**D**) The number of differentially expressed genes (log2|FC| > 2, FDR < 0.01) between Leg-DR and the two groups, Leg-DS and Carcass-DR. (**E**) CP genes enriched in Leg-DR, compared with Leg-DS. (**F**) CP genes enriched in Leg-DR, compared with Leg-DS and Carcass-DR.

Genes potentially related to cuticle proteins were selected for enriched expression in legs, based on comparison with carcass transcripts (Leg vs. Carcass). Among the 2699 up-regulated genes, 32 genes were identified as structural components of the cuticle, and 20 genes belonged to CP families, as determined by homologous sequence comparison with annotation of the Anopheles gambiae genome, including 16 genes in the CPR family (10 in the RR-1 family and 6 in the RR-2 family), and 4 genes *AsCPAP3-A1c*, *AsCPAP3-A1b*, *AsCPAP3-B*, and *AsCPAP3-D* (Figure 3A). Analysis of the Leg-DR vs. Carcass-DR and Leg-DS vs. Carcass−DS comparison groups showed differences and sharing of the CPs genes enriched in the legs. The results of Leg-DR vs. Carcass-DR showed that there were 20 genes belonging to the CP families, among which *AsCPF1* was a previously overlooked overexpressed gene, and 8 overexpressed genes encoding cuticle proteins (AsCPR5, *AsCPR38*, *AsCPR58*, *AsCPR129*, *AsCPR131*, *AsCPAP3-A1c*, *AsCPAP3-A1b*, and *AsCPAP3-B*) were not significantly expressed in Leg-DS vs. Carcass-DS (Figure 3B,C).

To further investigate the constitutive resistance of the legs, we compared transcriptome sequencing results from resistant *An. sinensis* legs (Leg-DR), susceptible *An. sinensis* legs (Leg-DS), and resistant *An. sinensis* carcasses (Carcass-DR). In these comparisons, 615 differentially expressed genes were identified (266 up-regulated and 349 down-regulated) between Leg-DR and Leg-DS, and 5329 differentially expressed genes (2512 up-regulated and 2817 down-regulated) between Leg-DR and Carcass-DR. In comparison of the two groups, there were 128 up-regulated genes and 95 down-regulated genes, indicating their potential association with the development of resistance phenotypes (Figure 3D).

Compared with Leg-DS, 10 genes related to cuticle were found among the 266 genes up-regulated by Leg-DR, of which 7 belonged to the CP families, including 4 CPR families (*AsCPR5*, *AsCPR58*, *AsCPR125*, and *AsCPR130*); 2 CPF families (*AsCPF1* and *AsCPF3*); and 1 CPAP family (*AsCPAP3-D*) (Figure 3E). Four genes explicitly related to cuticle structure were enriched in the legs of resistant *An. sinensis* among the 128 genes that were jointly up-regulated, namely, *AsCPR5*, *AsCPR58*, *AsCPF1*, and *AsCPF3* (Figure 3F).

### 2.4. Expression of Cuticle Protein Genes (CPGs)

**Figure 4:**
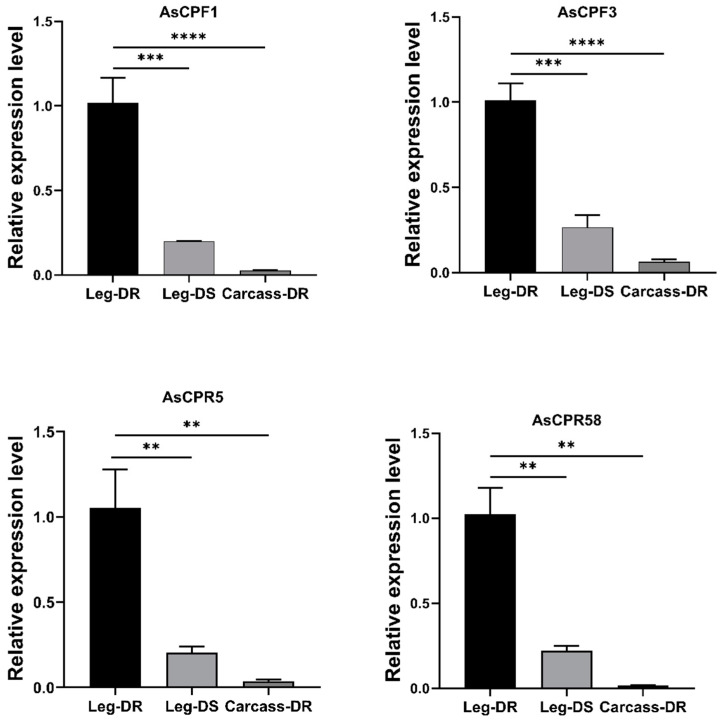
Relative expression levels of four CP genes in Leg-DR, Leg-DS, and Carcass-DR of *An. sinensis*. Asterisks indicate significant differences (Dunnett’s test, ** *p* < 0.01, *** *p* < 0.001, **** *p* < 0.0001).

RT-qPCR was used to assay expression of the four cuticle protein genes identified from screening, *AsCPR5*, *AsCPR58*, *AsCPF1*, and *AsCPF3*, the results showing high expression in Leg-DR compared to Leg-DS as well as Carcass-DR, which shared the same trend with the RNA-seq results (Figure 4).

**Figure 5:**
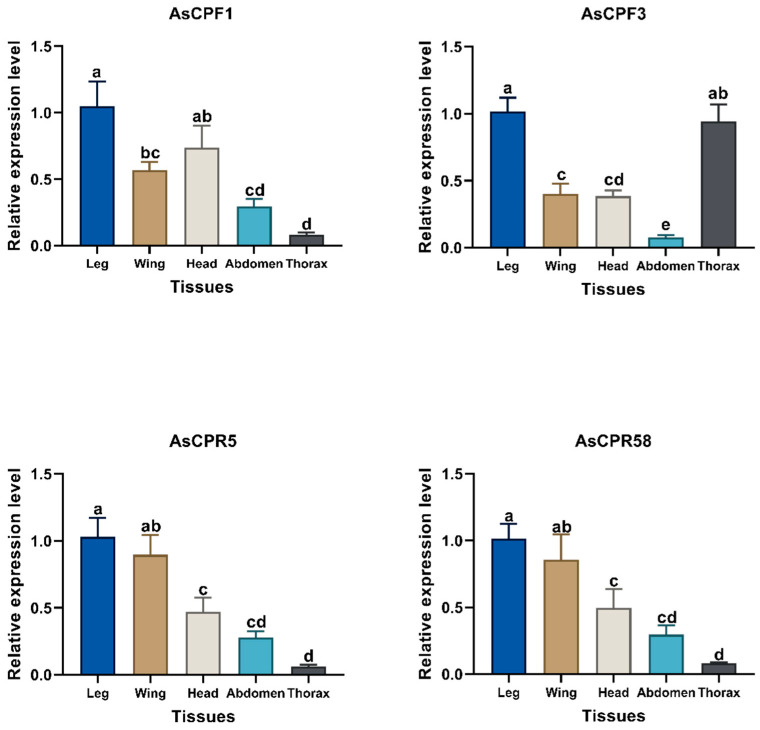
mRNA expression profile analysis of four CP genes in legs, wings, heads, abdomen, and thorax of DR *An. sinensis* (Duncan’s DMRT, and the same letter means no significant difference).

To further verify the high specificity of gene expression in the legs of *An. sinensis*, gene expression levels were assayed in various tissues of female DR *An. sinensis* approximately 3 days post-eclosion, specifically the legs, wings, heads, abdomen, and thorax. The four CP genes showed the highest expression levels in legs, followed by wings or heads, except for *AsCPF3*, which also had a high expression level in the abdomen (Figure 5).

### 2.5. Expression Response of CPGs Under Deltamethrin Exposure

**Figure 6:**
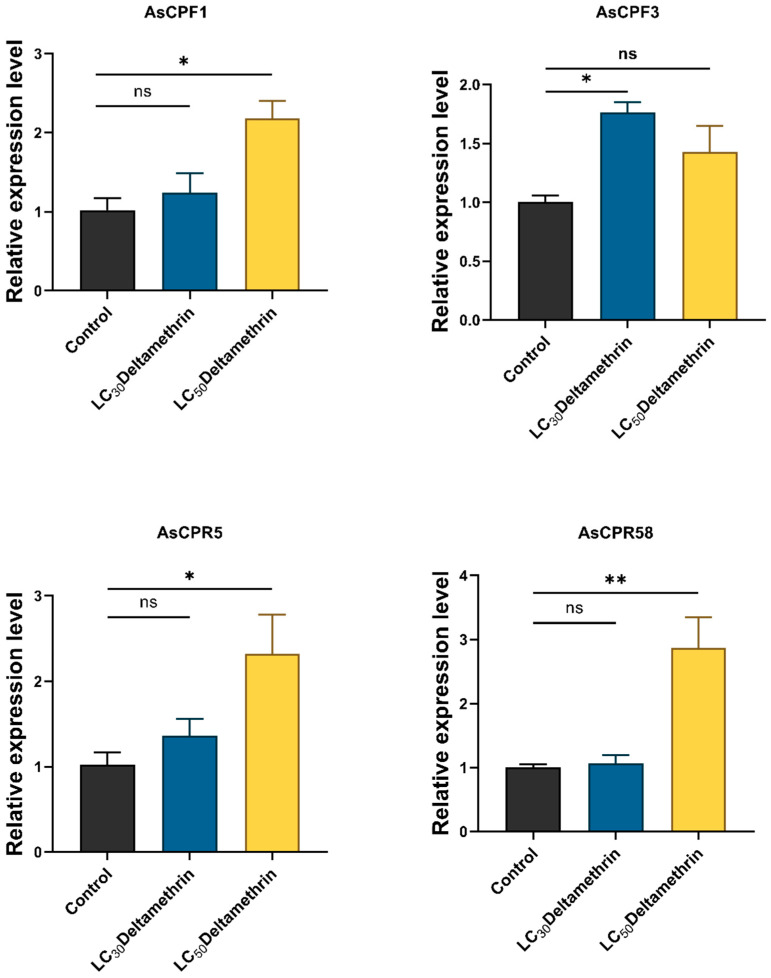
Relative expression level of four CP genes in the whole body of *An. sinensis* after deltamethrin treatment. Asterisks indicate significant differences (Dunnett’s test, * *p* < 0.05, ** *p* < 0.01, ns, not significant, *p* > 0.05).

The effects of deltamethrin on the transcript levels of *AsCPF1*, *AsCPF3*, *AsCPR5*, and *AsCPR58* genes were characterized by WHO bottle bioassays by treating 3 to 5 days female DS strain *An. sinensis* with deltamethrin at LC_30_ and LC_50_ concentrations. The results showed that *AsCPF1*, *AsCPR5*, and *AsCPR58* genes were significantly induced by deltamethrin treatment for 30 min, but no induction was observed at an LC_30_ concentration, with the exception of the *AsCPF3* gene (Figure 6).

### 2.6. Insecticide Resistance Bioassays and Examination of Cuticle Structure After RNAi

**Figure 7:**
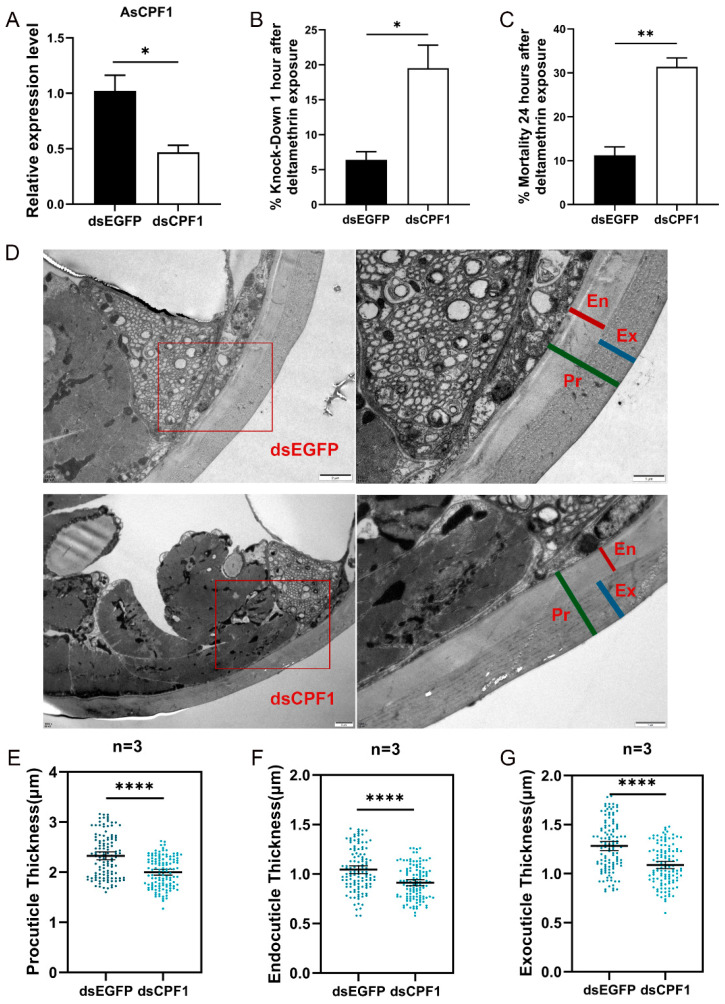
(**A**) Relative expression level of the *AsCPF1* gene in *An. sinensis* after injection of dsCPF1. (**B**,**C**) Knockdown rate and mortality of dsRNA-injected *An. sinensis* after exposure to 0.05% deltamethrin. (**D**) TEM observation of cuticle after dsRNA injection. (**E**–**G**) Cuticle thickness analysis by TEM. Each dot represents an individual cuticle location (each group was composed of 3 samples, and each sample has 40 points). Asterisks indicate significant differences (Student’s *t*-test, * *p* < 0.05, ** *p* < 0.01, **** *p* < 0.0001).

To characterize the role of *AsCPF1* in insecticide resistance, the expression of the gene was interfered with by microinjection of dsRNA, and RT-qPCR was used to measure gene silencing efficiency. Compared with the control group (dsEGFP injection), dsRNA-mediated gene silencing decreased the *AsCPF1* transcript level in the whole female mosquito by 54% (Figure 7A). Corresponding interference was also assayed on the remaining three genes, but satisfactory results were not obtained; therefore, only the function of *AsCPF1* was subsequently characterized. A WHO tube test was used to assay the effect of *AsCPF1* silencing on deltamethrin toxicity and demonstrated that mortality was higher in the experimental versus control group. Specifically, the mosquito knockdown rate was 6.6% after 1 h in the dsEGFP control injection group, versus 20% after 1 h in the dsCPF1 injection group (Figure 7B). After 24 h of recovery, the mortality rate of dsEGFP was 11.5%, while that of the dsCPF1 group was 31.7% (Figure 7C).

The cuticle ultrastructures of mosquito tarsus were characterized after RNA interference and showed that the procuticle thickness of the dsEGFP group (2.33 ± 0.43 μm) was thicker than that of the dsCPF1 group (2.00 ± 0.31 μm) (Figure 7D,E). The endocuticle and exocuticle thickness of the leg tarsus in the dsCPF1 group (0.91 ± 0.17 μm, 1.09 ± 0.20 μm) was thinner than in the dsEGFP group (1.04 ± 0.22 μm, 1.28 ± 0.25 μm) (Figure 7F,G). These data suggest that mosquitoes are more susceptible to deltamethrin after dsRNA knockdown of CPF1.

## 3. Discussion

The legs of mosquitoes play a key role in their life activities, which is reflected in their unique surface hydrophobic structure and their avoidance response to external chemical stimulation [21,22,23]. Insecticide-Treated Nets (ITNs) and Indoor Residual Spraying (IRS) are two key intervention measures on vector control. Residual sprays leave insecticides on the surface of objects, such as the interior walls of dwellings. When mosquitoes rest and stay on these surfaces, their legs are the primary contact parts and, therefore, may play an important role in the development of mosquito insecticide resistance [19,24].

The cuticles of insect legs are the initial physiological barrier for contact with external substances, and significantly influence the permeation of insecticides. The pathways responsible for the synthesis of this cuticular component have emerged as promising targets for vector control strategies [10]. In this study, we investigated the role of susceptible and resistant *An. sinensis* (DS and DR) in constitutive deltamethrin resistance based on the leg. We utilized a laboratory-propagated *An. sinensis* strain for the insecticide-susceptible experimental group, because field-caught mosquitoes typically have a degree of resistance to deltamethrin. By comparing the tarsus thickness of the legs between DS and DR *An. sinensis*, we first found that the tarsus cuticle of the DR *An. sinensis* was thicker than observed for DS, and the difference between the endocuticle seemed to be more pronounced. Similar thickening of the leg cuticle in resistant populations was found in other insects, such as the mosquitoes *Anopheles gambiae* and *Aedes aegypti*, cockroaches, and bed bugs [5,7,11,24,25]. In the study of specific transcripts in the *An. sinensis* leg, we found that a series of genes encoding cuticle proteins (CPs) were significantly upregulated in resistant *An. sinensis*, such as *AsCPR62*, *AsCPR127*, *AsCPR130*, *AsCPR143*, *AsCPAP3-A1b*, *AsCPAP3-A1c*, and *AsCPAP3-B*, which were also discovered in other resistant *Anopheles* strains. The gene *CPR62*, which is overexpressed in the legs of resistant *An. sinensis*, has been reported to be present in the legs of *Anopheles coluzzii*, and the overexpression of *CPAP3-A1b* and *CPAP3-A1c* are associated with resistance to permethrin and deltamethrin [19,26]. Comparing transcription data between susceptible and resistant legs, we found that the gene *AsCPF3* was overexpressed in resistant legs of *An. gambiae*, primarily localizing to the exocuticle, thus suggesting that this gene could be used as a candidate marker for resistance [27].

At present, there are gaps in understanding the function of CPGs in insect resistance. We have screened four cuticle protein-related genes that are overexpressed in the legs of the DR strain, specifically *AsCPF1*, *AsCPF3*, *AsCPR5,* and *AsCPR58*. The expression profiles of the related genes were further analyzed, and their effects on deltamethrin in *An. sinensis* were evaluated.

CPR and CPF are two important gene families which encode cuticle proteins in mosquitoes. One of the distinctive characteristic of the CPR family of genes is that they contain the Rebers and Riddiford (RR) consensus sequence, which binds to chitin to enhance the stability of cuticle structures [28]. CPFs are a small family of proteins defined by 51 amino acid motifs, containing 44 conserved regions of amino acids, and do not exhibit the ability to bind to chitin [27,29]. It has been reported that some cuticle proteins are differentially expressed between insecticide-susceptible and insecticide-resistant mosquito strains. Among them, CPR family members, as one of the ubiquitous cuticular proteins, have been shown to enhance insecticide resistance of mosquitoes to insecticides by changing the thickness of the cuticle [14,30,31,32]. It is noteworthy that although current research on the relationship between the CPF gene family and insecticide resistance is scarce, studies have shown that CPF and the similar CPFL family proteins are highly expressed in insecticide resistant populations [27,29,33]. Preliminary results indicate that the expression levels of *AsCPR5*, *AsCPR58*, *AsCPF1*, and *AsCPF3* in the legs of the DR *An. sinensis* were elevated compared to both the carcasses of the DR *An. sinensis* and the legs of the DS *An. sinensis*. Among them, expression of the CPR5 protein in *Culex pipiens pallens* has been shown to be modulated by miR-932, thereby influencing resistance levels [34]. Subsequently, we examined the expression profiles of the genes across different tissues. The findings revealed high expression levels in wings and heads, which may also be related to the function of helping insects escape harmful environments [35]. High expression of the *AsCPF3* gene was observed in the abdomen. Given that the abdomen has a detoxification system and reproductive organs, elevated expression of the *AsCPF3* gene may suggest physiological functions outside the cuticle structure, thus highlighting its multifaceted importance in insect biology.

Studies have shown that insecticides can induce the expression of CPGs, which in turn enhance insect resistance by increasing cuticle thickness and density [33]. Therefore, we used deltamethrin to treat *An. sinensis* mosquitoes and determined that the expression of these four CP genes significantly increased, indicating their potential involvement in the mosquito response to and tolerance of deltamethrin. To further validate the role of the screened CPGs in the tolerance of *An. sinensis* to deltamethrin, we used RNA interference (RNAi) technology to silence the *AsCPF1* gene. RNA interference was performed on the other three candidate genes, but only *AsCPF1* had efficient interference. There was a significant variation in interference efficiency among the different genes across various species and even within the same species. For those genes with poor interference efficiency, we speculate that this may be due to insufficient intracellular transport efficiency of dsRNA after injection, and the majority of the dsRNA failing to successfully escape from endosomes, thus preventing their conversion into siRNA and a resulting failure to exert their expected interfering effects [36,37,38]. The results showed that dsRNA injection of *AsCPF1* significantly inhibited the expression of this gene in deltamethrin-resistant populations. Resistance assays performed using the WHO tube bioassay after *AsCPF1* was knocked down showed that insect mortality increased, thus indicating a significant increase in the susceptibility of *An. sinensis* to deltamethrin. Similar results have been reported when silencing the cuticle protein genes of *Culex pipiens pallens*, such as *CPR47*, *CPR63*, and *CPLCG5* [12,30].

Silencing of *AsCPF1* resulted in a thinner endocuticle and exocuticle, suggesting that increasing the thickness of the cuticle contributes to cuticular resistance, and may be one of the functions of *AsCPF1*, like most CPRs proteins. It has been reported that CPF protein has similar structural characteristics to lipid carrier protein [39]; therefore, we might speculate that the CPF protein may have a function similar to lipid transport. By enhancing its binding ability to insecticides, it provides more time for the detoxification metabolism process in mosquitoes and reduces the direct effect of insecticides on target sites, thereby enhancing the resistance of mosquitoes. Although the CPF and CPLCP family proteins lack a domain that binds directly to chitin, some studies suggest that these CPs may interact with other structural proteins in the insect cuticle, thereby contributing to the formation of a harder epidermal matrix in insects [30]. However, the detail mechanism and function pathway need to be further explored and verified.

In conclusion, this study preliminarily identified and analyzed the enrichment of cuticular proteins in the legs of *An. sinensis*. We found a significant abundance of previously overlooked CP genes, which were enriched and expressed in the legs, thus providing new insights into insecticide resistance management for mosquito populations.

## 4. Materials and Methods

### 4.1. Mosquito Rearing and Collection

The deltamethrin-susceptible *An. sinensis* line used in this study was initially collected in Jiangsu, China, and has been colonized in the laboratory for more than 40 years. The strain has not been exposed to insecticides during the past decades [40]. In 2023, blood-engorged female anopheline mosquitoes were collected in Huai’an City, Jiangsu, China, where previous data showed that *An. sinensis* was the sole anopheline species present and exhibited high resistant to deltamethrin [40]. The samples brought back to the laboratory were used to confirm the species of the hatched eggs based on key morphological features [41]. A group of 3 to 5 days female *An. sinensis* from the first generation (F1) were selected for subsequent experiments. The mosquito rearing conditions were as follows: temperature (25 ± 1) °C, relative humidity (75 ± 5%), light intensity 12 h/d, larvae fed with tropical fish diet, and adults fed with 5% glucose.

### 4.2. Insecticide Resistance Bioassay of Field Anopheles sinensis

F1 female *An. sinensis* were collected 3 to 5 days and were exposed for 1 h to 0.05% deltamethrin-treated film (Chinese Center for Disease Control and Prevention, Beijing, China) using the WHO-recommended tube test method [42]. After 24 h of recovery, the mortality rate was recorded. Each tube of about 20 mosquitoes, repeat five times (resistant: mortality < 90%; possible resistant: 90% ≥ mortality ≤ 98%; susceptible: > 98%).

### 4.3. Transmission Electron Microscopy (TEM) and Image Analysis

The thickness of the cuticle on the tarsus of mosquito legs was measured using Transmission Electron Microscopy (TEM) (Wuhan MISP Bio-technology CO, LTD). Specifically, 3 to 5 days female *An. sinensis* were selected from the laboratory deltamethrin-susceptible strain (DS) and the field F1 deltamethrin-resistant population (DR), and individuals of similar size were selected by measuring the wing length of each individual [43]. Eight *An. sinensis* from DS and DR were randomly selected as independent biological replicates, and their tarsi (tarsus I) of the midleg were dissected for ultrathin sections. The specimens were preserved in 2.5% glutaraldehyde solution (PH 7.4) for 2 h. Then, washing three times with 0.1 M phosphate buffer (pH 7.2), the samples were fixed in 1% osmium tetroxide at 4 °C for 2 h, and samples were gradient dehydrated in a graded series of ethanol. Subsequently, the samples were embedded in Epon-epoxy resin for infiltration and placed in molds for polymerization. After semi-thin sections were used for orientation, ultra-thin sections were produced and collected for microstructural analysis. These sections were then counterstained with 3% uranyl acetate and 2.7% lead citrate. Finally, the sections were observed using a JEM1400 TEM.

Figure S3 in the Additional File shows a mosquito leg image obtained by scanning electron microscopy. The thickness of the cuticle was examined using Nano Measurer 1.2 software. The cross-sections were imaged by TEM, and the exocuticle, endocuticle, and procuticle measurements were obtained from 8 tarsi in each group, with 40 measurement points per tarsus.

### 4.4. Transcriptomic Analysis

#### 4.4.1. RNA Extraction, RNA-Seq Library Preparation and Sequencing

Samples for RNA extraction were divided into four types: the carcass of DS *An. sinensis*, the leg of DS *An. sinensis*, the carcass of DR *An. sinensis*, and the leg of DR *An. sinensis*. The legs of 3 to 5 days DS and DR *An. sinensis* were separated under a microscope (four biological replicates for each type of sample). For the carcass samples of DS and DR *An. sinensis*, 10 mosquitoes were pooled in each tube, and for leg samples, extractions 30 to 50 mosquitoes were used. The collected samples were immediately treated with liquid nitrogen for 5 min and stored in a −80 °C freezer.

Total RNA was extracted using a Vazyme kit (R711). RNA purity and quantification were determined using a NanoDrop 2000 spectrophotometer (Thermo Scientific, Wilmington, DE, USA), and RNA integrity was assessed using an Agilent 2100 Bioanalyzer (Agilent Technologies, Santa Clara, CA, USA). Transcriptome libraries were constructed using a VAHTS Universal V6 RNA-seq Library Prep kit.

#### 4.4.2. RNA Sequencing and Differentially Expressed Gene Analysis

The library sequencing used the Illumina Novaseq 6000 sequencing platform to produce 150 bp paired-end reads. The low-quality reads in the original reads were removed using Fastp software to obtain clean reads for subsequent data analysis [44]. HISAT2 software was used for gene expression (FPKM) calculation, and HTSeq-count was used to obtain the read counts of each gene [45,46,47]. Principal Component Analysis (PCA) was performed using R (v 3.2.0) to evaluate sample biological repeats. DESeq2 software was used to analyze the differentially expressed genes, with a threshold of q value < 0.01 and fold change > 2 [48]. Based on the hypergeometric distribution, the entries with significant enrichment of DEGs were screened across different groups. Graphics were performed using R (v 3.2.0) to show the expression patterns of genes in different samples and groups.

### 4.5. Quantitative PCR for RNA-seq Data Validation

Fluorescence quantitative analysis was performed on the candidate cuticle protein genes to verify the results of RNA-seq data. The extraction and determination methods of RNA were the same as above. The first-strand cDNA was synthesized using a reverse transcription kit HiScript^®^ II 1st Strand cDNA Synthesis Kit (+gDNA wiper) (Vazyme Biotechnology Co., Ltd., Nanjing, China). The specific RT-qPCR primers for the CP genes of *An. sinensis* were designed using Premier 5.0 software, with *AsS7* as the internal reference gene (Table 1). The reaction system is as follows: 5 μL of 2× ChamQ Universal SYBR qPCR Master Mix, 0.2 μL 10 μmol/L forward and reverse primers, 1 μL cDNA template, and 3.6 μL RNase-free water. The reaction program was pre-denaturation at 95 °C for 30 s, denaturation at 95 °C for 10 s, and extension at 60 °C for 30 s (40 cycles).

### 4.6. Quantitative Analysis of CP Genes in Different Tissues of Anopheles sinensis

The legs, wings, heads, abdomen, and thorax were collected from 3 to 5 days female DR *An. sinensis* that had not been fed blood. Total RNA extraction, cDNA synthesis, and RT-qPCR experiments were carried out as described above on each group of 15 mosquitoes, with at least three biological replicates.

### 4.7. Gene Functional Verification

#### 4.7.1. Response of CP Gene Expression to Deltamethrin Exposure

Based on the initial test, female DS *An. sinensis* exhibited an LC_50_ of 2.39 mg/L for deltamethrin, as measured by the WHO bottle bioassay [49]. The concentrations of deltamethrin at LC_30_ (1.71 mg/L) and LC_50_ (2.39 mg/L) were used to determine the response of *An. sinensis* to deltamethrin for 30 m, with ethyl alcohol as the control group. The experimental methods are the same as in Section 4.5. Each group was compsed of 10 mosquitoes, with at least three biological replicates.

#### 4.7.2. dsRNA Synthesis and Microinjection

The dsRNA template with T7 promoter sequences (TAATACGACTCACTATAGG) at both ends was synthesized by PCR. Primers were designed using Premier 5.0 software (Table 1). The PCR was performed with the following steps: pre-denaturation at 95 °C for 3 min, followed by 30 cycles of denaturation at 95 °C for 15 s, annealing at 55 °C for 15 s, and extension at 72 °C for 20 s. After the cycles, a final extension was done at 72 °C for 5 min. ds*AsCPF1* and control dsEGFP were synthesized using the P1700 T7 RiboMAXtm Express RNAi Kit (Promega, Fitchburg, WI, USA). The quality and concentration of dsRNA were determined using a 2% gel imager.

A Nanoject III microinjector (Drummond, Broomall, PA, USA) was used to inject 210 ng of ds RNA into the thorax between the second and third leg segments of 1-day-old female DR *An. sinensis*. The injected adult mosquitoes were collected 5 days later for subsequent assays. The experimental methods are the same as in Section 4.5. Each group consists of 10 mosquitoes, with at least three biological replicates.

#### 4.7.3. Insecticide Resistance Bioassays After RNAi

The WHO tube test method (0.05% deltamethrin) was conducted 5 days after dsRNA injection, and all treatments were repeated three times. The mosquitoes were transferred back to holding tubes for 1 h, and the mortality rate was calculated after 24 h of recovery, using the method described above.

#### 4.7.4. Examination of Cuticle Structure After RNAi

After dsRNA injection, three middle legs were randomly selected as three independent biological replicates, and the sample processing and image analysis were conducted as described above. The cuticle thickness measurements were obtained from 3 tarsi in each group, with 40 measurement points per tarsus.

### 4.8. Statistical Analysis

The 2^−ΔΔCt^ method was used to analyze the RT-qPCR results. SPSS 25.0 software was used to calculate the mortality rate of *An. sinensis* in the bioassay experiment. Data analysis was performed using Student’s *t*-test and one-way ANOVA. Data were expressed as Mean ± SD for TEM analysis and Mean ± SEM for all other results. To verify the data at the transcriptional level, ANOVA followed by Dunnett’s test was performed. To evaluate the differences in the expression level of the candidate genes in different tissues and under two concentrations of deltamethrin stress, ANOVA followed by Duncan’s DMRT was utilized.

## Figures and Tables

**Table 1:** Primers for PCR, dsRNA and RT-qPCR.

Primer Sequence (5′-3′)	
For RT-qPCR	
*AsS7*-F	AAGTTCTCCGGCAAGCATGT
*AsS7*-R	GGTCGCTTCTGCTTGTTGG
*AsCPF1*-F	CCCATGATGGAACCGTCTCG
*AsCPF1*-R	GTGATGCGGGTGTCCGACTT
*AsCPF1* ds-F	CATTCAAGTTCGTCGTCTTCCTGG
*AsCPF1* ds-R	CCTGCGAGATGGTGCTGTAGCT
*AsCPF3*-F	CGTCTGTCAGCAAGTCCGATGT
*AsCPF3*-R	CGGCGTAAGCGTGATGAGC
*AsCPR5*-F	GGAGATGTTGTCCAGGGATCGTA
*AsCPR5*-R	GTTGTGCGGGTCAGCAGTGTAG
*AsCPR58*-F	GAGCCTGTCGTACACGTTGCC
*AsCPR58*-R	CATAGTATCCATCGTGGTAGTCA
For *AsCPF1* dsRNA synthesis	
T7 EGFP ds-F GGATCCTAATACGACTCACTATAGGTGCCCGAAGGTTATGT	
T7 EGFP ds-R GGATCCTAATACGACTCACTATAGGTGCCGAGTGTAATCCC	
T7 *AsCPF1* ds-F GGATCCTAATACGACTCACTATAGGACACCCGCATCACCAACGAG	
T7 *AsCPF1* ds-R GGATCCTAATACGACTCACTATAGGGGCATAATGGGCATGAGCATC	

## Data Availability

The raw sequence reads obtained from RNA-seq were submitted to NCBI Sequence Read Archive (SRA) under BioProject PRJNA1224403.

## Supplementary Materials

The following supporting information can be downloaded at https://www.mdpi.com/article/10.3390/ijms26052182/s1.

## References

[1] Zhao Y.-Q., Tang Y.-Y., Hu J.-P., Huang Y.-Z., Wan K., Zhang M.-H., Li J.-L., Zhu G.-D., Tang J.-X. (2024). An Aquaporin and an Aquaglyceroporin Have Roles in Low Temperature Adaptation of Mosquitoes (*Anopheles sinensis*). Insect Sci..

[2] Van den Berg H., da Silva Bezerra H.S., Al-Eryani S., Chanda E., Nagpal B.N., Knox T.B., Velayudhan R., Yadav R.S. (2021). Recent Trends in Global Insecticide Use for Disease Vector Control and Potential Implications for Resistance Management. Sci. Rep..

[3] Bhatt S., Weiss D.J., Cameron E., Bisanzio D., Mappin B., Dalrymple U., Battle K., Moyes C.L., Henry A., Eckhoff P.A. (2015). The Effect of Malaria Control on *Plasmodium falciparum* in Africa between 2000 and 2015. Nature.

[4] Akoton R., Sovegnon P.M., Djihinto O.Y., Medjigbodo A.A., Agonhossou R., Saizonou H.M., Tchigossou G.M., Atoyebi S.M., Tossou E., Zeukeng F. (2023). Vectorial Competence, Insecticide Resistance in *Anopheles funestus* and Operational Implications for Malaria Vector Control Strategies in Benin Republic. Malar. J..

[5] Jacobs E., Chrissian C., Rankin-Turner S., Wear M., Camacho E., Broderick N.A., McMeniman C.J., Stark R.E., Casadevall A. (2023). Cuticular Profiling of Insecticide Resistant *Aedes aegypti*. Sci. Rep..

[6] Meng L.-W., Yuan G.-R., Chen M.-L., Zheng L.-S., Dou W., Peng Y., Bai W.-J., Li Z.-Y., Vontas J., Wang J.-J. (2023). Cuticular Competing Endogenous RNAs Regulate Insecticide Penetration and Resistance in a Major Agricultural Pest. BMC Biol..

[7] Cai T., Wang X., Liu B., Zhao H., Liu C., Zhang X., Zhang Y., Gao H., Schal C., Zhang F. (2024). A Cuticular Protein, *BgCPLCP1*, Contributes to Insecticide Resistance by Thickening the Cockroach Endocuticle. Int. J. Biol. Macromol..

[8] Balabanidou V., Grigoraki L., Vontas J. (2018). Insect Cuticle: A Critical Determinant of Insecticide Resistance. Curr. Opin. Insect Sci..

[9] Yan Z., Tong X., Xiong G., Yang W., Lu K., Yuan Y., Han M., Hu H., Wei W., Dai F. (2022). A Blueprint of Microstructures and Stage-Specific Transcriptome Dynamics of Cuticle Formation in Bombyx Mori. Int. J. Mol. Sci..

[10] Ren Y., Li Y., Ju Y., Zhang W., Wang Y. (2023). Insect Cuticle and Insecticide Development. Arch. Insect Biochem. Physiol..

[11] Yahouédo G.A., Chandre F., Rossignol M., Ginibre C., Balabanidou V., Mendez N.G.A., Pigeon O., Vontas J., Cornelie S. (2017). Contributions of Cuticle Permeability and Enzyme Detoxification to Pyrethroid Resistance in the Major Malaria Vector Anopheles Gambiae. Sci. Rep..

[12] Sun X., Guo J., Ye W., Guo Q., Huang Y., Ma L., Zhou D., Shen B., Sun Y., Zhu C. (2017). Cuticle Genes *CpCPR63* and *CpCPR47* May Confer Resistance to Deltamethrin in *Culex pipiens pallens*. Parasitol. Res..

[13] Soh L.-S., Veera Singham G. (2021). Cuticle Thickening Associated with Fenitrothion and Imidacloprid Resistance and Influence of Voltage-Gated Sodium Channel Mutations on Pyrethroid Resistance in the Tropical Bed Bug, *Cimex Hemipterus*. Pest. Manag. Sci..

[14] Xu Y., Xu J., Zhou Y., Li X., Meng Y., Ma L., Zhou D., Shen B., Sun Y., Zhu C. (2022). CPR63 Promotes Pyrethroid Resistance by Increasing Cuticle Thickness in *Culex pipiens pallens*. Parasit. Vectors.

[15] Tan S., Li G., Guo H., Li H., Tian M., Liu Q., Wang Y., Xu B., Guo X. (2022). Identification of the Cuticle Protein *AccCPR2* Gene in *Apis cerana cerana* and Its Response to Environmental Stress. Insect Mol. Biol..

[16] Zhou D., Duan B., Sun Y., Ma L., Zhu C., Shen B. (2017). Preliminary Characterization of Putative Structural Cuticular Proteins in the Malaria Vector *Anopheles sinensis*: Cuticular Proteins in *Anopheles sinensis*. Pest. Manag. Sci..

[17] Zheng J., Wu P., Huang Y., Zhang Y., Qiu L. (2024). Identification of Insect Cuticular Protein Genes *LCP17* and *SgAbd5* from *Helicoverpa armigera* and Evaluation Their Roles in Fenvalerate Resistance. Pestic. Biochem. Physiol..

[18] Kefi M., Balabanidou V., Sarafoglou C., Charamis J., Lycett G., Ranson H., Gouridis G., Vontas J. (2023). ABCH2 Transporter Mediates Deltamethrin Uptake and Toxicity in the Malaria Vector *Anopheles coluzzii*. PLoS Pathog..

[19] Kefi M., Charamis J., Balabanidou V., Ioannidis P., Ranson H., Ingham V.A., Vontas J. (2021). Transcriptomic Analysis of Resistance and Short-Term Induction Response to Pyrethroids, in *Anopheles coluzzii* Legs. BMC Genom..

[20] Matthews B.J., McBride C.S., DeGennaro M., Despo O., Vosshall L.B. (2016). The Neurotranscriptome of the *Aedes aegypti* Mosquito. BMC Genom..

[21] Dennis E.J., Goldman O.V., Vosshall L.B. (2019). *Aedes aegypti* Mosquitoes Use Their Legs to Sense DEET on Contact. Curr. Biol..

[22] Sparks J.T., Vinyard B.T., Dickens J.C. (2013). Gustatory Receptor Expression in the Labella and Tarsi of *Aedes aegypti*. Insect Biochem. Mol. Biol..

[23] Wu C.W., Kong X.Q., Wu D. (2007). Micronanostructures of the Scales on a Mosquito’s Legs and Their Role in Weight Support. Phys. Rev. E.

[24] Balabanidou V., Kefi M., Aivaliotis M., Koidou V., Girotti J.R., Mijailovsky S.J., Juárez M.P., Papadogiorgaki E., Chalepakis G., Kampouraki A. (2019). Mosquitoes Cloak Their Legs to Resist Insecticides. Proc. R. Soc. B..

[25] Lilly D.G., Latham S.L., Webb C.E., Doggett S.L. (2016). Cuticle Thickening in a Pyrethroid-Resistant Strain of the Common Bed Bug, *Cimex Lectularius L*. (Hemiptera: Cimicidae). PLoS ONE.

[26] Saizonou H., Impoinvil L.M., Derilus D., Omoke D., Okeyo S., Dada N., Corredor C., Mulder N., Lenhart A., Ochomo E. (2024). Transcriptomic Analysis of *Anopheles gambiae* from Benin Reveals Overexpression of Salivary and Cuticular Proteins Associated with Cross-Resistance to Pyrethroids and Organophosphates. BMC Genom..

[27] Vannini L., Reed T.W., Willis J.H. (2014). Temporal and Spatial Expression of Cuticular Proteins of *Anopheles Gambiae* Implicated in Insecticide Resistance or Differentiation of M/S Incipient Species. Parasites Vectors.

[28] Vannini L., Willis J.H. (2017). Localization of RR-1 and RR-2 Cuticular Proteins within the Cuticle of *Anopheles gambiae*. Arthropod Struct. Dev..

[29] Tang P.-A., Hu H.-Y., Du W.-W., Jian F.-J., Chen E.-H. (2023). Identification of Cuticular Protein Genes and Analysis of Their Roles in Phosphine Resistance of the Rusty Grain Beetle *Cryptolestes ferrugineus*. Pestic. Biochem. Physiol..

[30] Huang Y., Guo Q., Sun X., Zhang C., Xu N., Xu Y., Zhou D., Sun Y., Ma L., Zhu C. (2018). *Culex pipiens pallens* Cuticular Protein *CPLCG5* Participates in Pyrethroid Resistance by Forming a Rigid Matrix. Parasites Vectors.

[31] He C., Liang J., Yang J., Xue H., Huang M., Fu B., Wei X., Liu S., Du T., Ji Y. (2023). Over-Expression of CP9 and CP83 Increases Whitefly Cell Cuticle Thickness Leading to Imidacloprid Resistance. Int. J. Biol. Macromol..

[32] Zoh M.G., Bonneville J.-M., Laporte F., Tutagata J., Sadia C.G., Fodjo B.K., Mouhamadou C.S., McBeath J., Schmitt F., Horstmann S. (2023). Deltamethrin and Transfluthrin Select for Distinct Transcriptomic Responses in the Malaria Vector *Anopheles gambiae*. Malar. J..

[33] Chen E.-H., Hou Q.-L. (2021). Identification and Expression Analysis of Cuticular Protein Genes in the Diamondback Moth, *Plutella xylostella* (Lepidoptera: Plutellidae). Pestic. Biochem. Physiol..

[34] Liu B., Tian M., Guo Q., Ma L., Zhou D., Shen B., Sun Y., Zhu C. (2016). MiR-932 Regulates Pyrethroid Resistance in *Culex pipiens pallens* (Diptera: Culicidae). J. Med. Entomol..

[35] Xu Y., Yang X., Sun X., Li X., Liu Z., Yin Q., Ma L., Zhou D., Sun Y., Shen B. (2020). Transcription Factor FTZ-F1 Regulates Mosquito Cuticular Protein *CPLCG5* Conferring Resistance to Pyrethroids in *Culex pipiens pallens*. Parasit. Vectors.

[36] Shukla J.N., Kalsi M., Sethi A., Narva K.E., Fishilevich E., Singh S., Mogilicherla K., Palli S.R. (2016). Reduced Stability and Intracellular Transport of dsRNA Contribute to Poor RNAi Response in Lepidopteran Insects. RNA Biol..

[37] Yoon J.-S., Gurusamy D., Palli S.R. (2017). Accumulation of dsRNA in Endosomes Contributes to Inefficient RNA Interference in the Fall Armyworm, *Spodoptera frugiperda*. Insect Biochem. Mol. Biol..

[38] Zhu K.Y., Palli S.R. (2020). Mechanisms, Applications, and Challenges of Insect RNA Interference. Annu. Rev. Entomol..

[39] Papandreou N.C., Iconomidou V.A., Willis J.H., Hamodrakas S.J. (2010). A Possible Structural Model of Members of the CPF Family of Cuticular Proteins Implicating Binding to Components Other than Chitin. J. Insect Physiol..

[40] Li Y., Li Y., Wang G., Li J., Zhang M., Wu J., Liang C., Zhou H., Tang J., Zhu G. (2022). Differential Metabolome Responses to Deltamethrin between Resistant and Susceptible *Anopheles sinensis*. Ecotoxicol. Environ. Saf..

[41] Rueda L.M., Kim H.-C., Chong S.-T., Klein T.A., Debboun M. (2017). Biosurveillance and Morphological Variations of Larvae and Pupae of Common Malaria Vectors, *Anopheles (Anopheles) hyrcanus* Group Species in the Republic of Korea. US Army Med. Dep. J..

[42] World Health Organization Standard Operating Procedure for Testing Insecticide Susceptibility of Adult Mosquitoes in WHO Tube Tests. https://www.who.int/publications/i/item/9789240043831.

[43] Koella J.C., Lyimo E.O. (1996). Variability in the Relationship between Weight and Wing Length of *Anopheles Gambiae* (Diptera: Culicidae). J. Med. Entomol..

[44] Chen S., Zhou Y., Chen Y., Gu J. (2018). Fastp: An Ultra-Fast All-in-One FASTQ Preprocessor. Bioinformatics.

[45] Kim D., Langmead B., Salzberg S.L. (2015). HISAT: A Fast Spliced Aligner with Low Memory Requirements. Nat. Methods.

[46] Roberts A., Trapnell C., Donaghey J., Rinn J.L., Pachter L. (2011). Improving RNA-Seq Expression Estimates by Correcting for Fragment Bias. Genome Biol..

[47] Anders S., Pyl P.T., Huber W. (2015). HTSeq—A Python Framework to Work with High-Throughput Sequencing Data. Bioinformatics.

[48] Love M.I., Huber W., Anders S. (2014). Moderated Estimation of Fold Change and Dispersion for RNA-Seq Data with DESeq2. Genome Biol..

[49] World Health Organization Standard Operating Procedure for Testing Insecticide Susceptibility of Adult Mosquitoes in WHO Bottle Bioassays. https://www.who.int/publications/i/item/9789240043770.

