# Supplementary material for: A Leg Cuticle Protein Enhances the Resistance of Anopheles sinensis Mosquitoes to Deltamethrin
Source: Int J Mol Sci. 2025 Feb 28;26(5):2182. doi: 10.3390/ijms26052182 (PMC11900137; doi:10.3390/ijms26052182)
Supplement: Supplementary file 1 [file ijms-26-02182-s001.zip › ijms-3466419-supplementary.pdf]

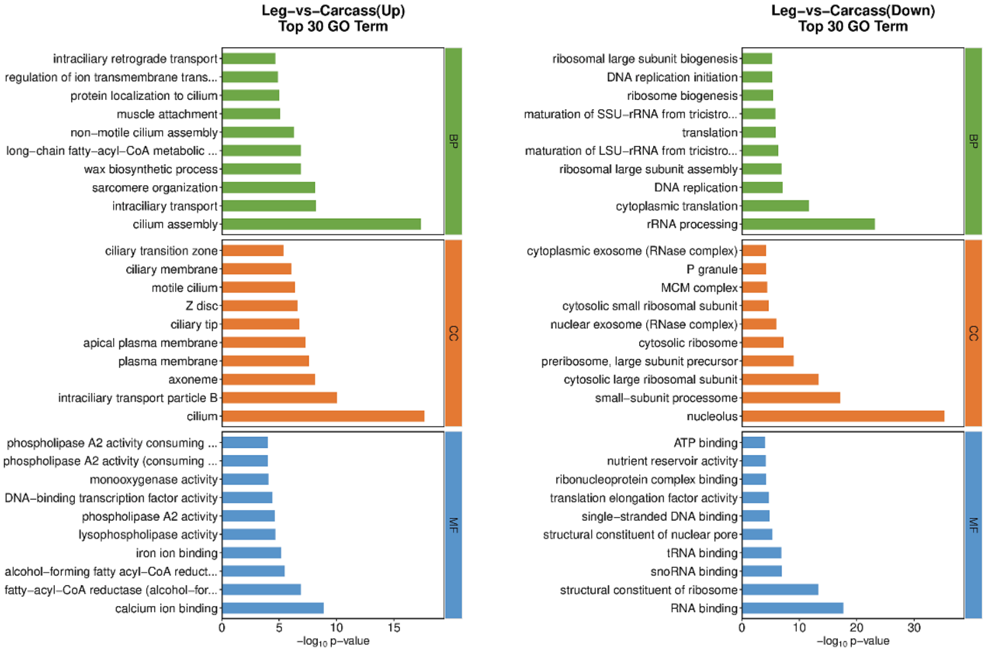

Figure S1. GO enrichment histogram in comparison of Leg and Carcass in *An.sinensis*

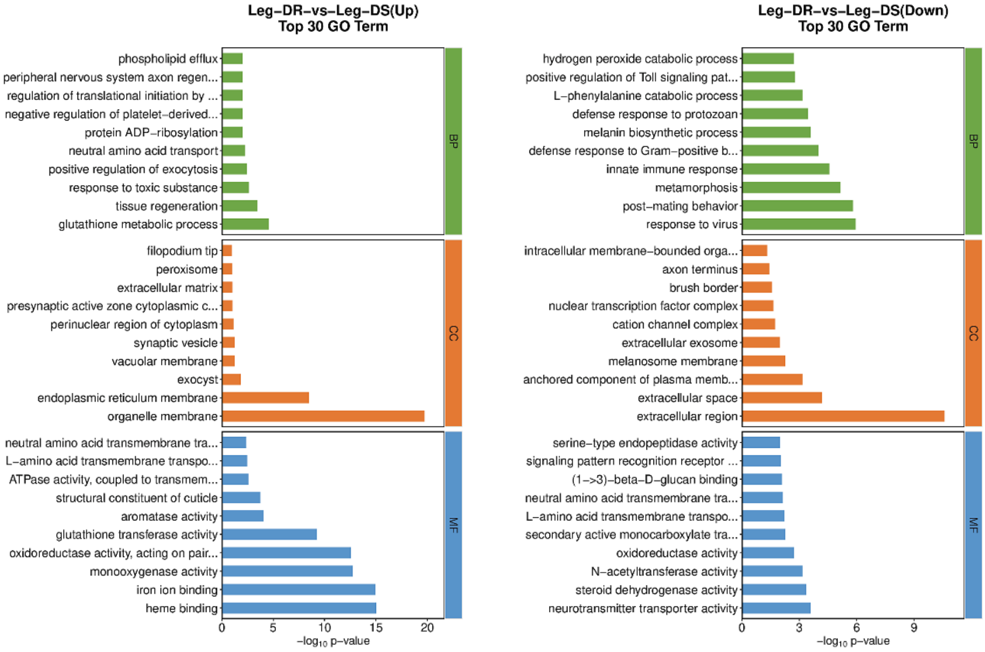

Figure S2. GO enrichment histogram in comparison of Leg–DR and Leg–DS in *An.sinensis*

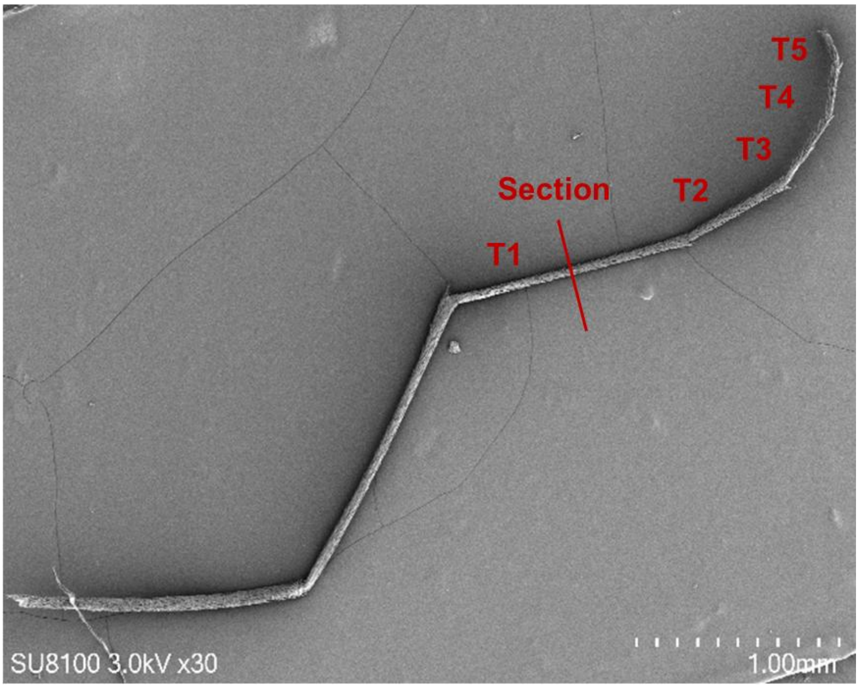

Figure S3. Illustration of the section at *An. sinensis* tarsus segment 1 (T1–T5 = five tarsal segments)
